# Changing characteristics of *S. aureus* bacteremia caused by PVL-negative, MRSA strain over 11 years

**DOI:** 10.1038/s41598-021-95115-2

**Published:** 2021-08-03

**Authors:** Eunmi Yang, Eunsil Kim, Hyemin Chung, Yun Woo Lee, Seongman Bae, Jiwon Jung, Min Jae Kim, Yong Pil Chong, Sung-Han Kim, Sang-Ho Choi, Sang-Oh Lee, Yang Soo Kim

**Affiliations:** 1 Division of Infectious Diseases, Asan Medical Center, University of Ulsan College of Medicine, 88, Olympic-ro43-gil, Songpa-gu, Seoul, 05505 Republic of Korea; 2 Center for Antimicrobial Resistance and Microbial Genetics, University of Ulsan College of Medicine, Seoul, Republic of Korea

**Keywords:** Medical research, Pathogenesis

## Abstract

Community-acquired methicillin-resistant *Staphylococcus aureus* (MRSA) has emerged as an important cause of infection. We conducted a longitudinal study to evaluate changes in clinical and microbiological characteristics as well as outcomes of sequence type (ST) 72 MRSA bacteremia. We reviewed adult patients enrolled in a prospective cohort with ST72 MRSA bacteremia from August 2008 to December 2018 at Asan Medical Center, Seoul, South Korea. Changes in clinical characteristics, outcomes, and microbiological characteristics of patients over time were evaluated. Generalized linear and linear regression models were used to evaluate changes. Of the 1,760 isolates, 915 (62%) were MRSA bacteremia and 292 (31.9%) were ST72 MRSA. During the study period, the relative risk (RR) of MRSA bacteremia decreased annually by 3.7%; however, among MRSA bacteremia, RR of ST72 MRSA increased annually by 8.5%. Vancomycin minimum inhibitory concentration (MIC) decreased over the study period. Metastatic infection, persistent bacteremia, and recurrence of bacteremia within 12 weeks decreased significantly. There were no significant changes in 30-d and 12-week mortality. Antibiotic susceptibility of ST72 MRSA was evaluated, and the resistance rate to erythromycin decreased significantly. ST72 MRSA incidence increased annually; its vancomycin MIC and erythromycin resistance rate decreased over the 11 years.

## Introduction

Methicillin-resistant *Staphylococcus aureus* (MRSA) is a leading cause of community- and healthcare-associated infections. Hospital-acquired (HA) MRSA and community-acquired (CA) MRSA clones are genetically distinct and their distribution varies in different regions^1^. However, in recent years, CA-MRSA has spread and emerged as an important cause of healthcare-associated infection^2,3^. USA300-sequence type (ST)8, a highly prominent Panton-Valentine leucocidin (PVL)-positive CA-MRSA, is the dominant CA-MRSA in North America and is progressively increasing in nosocomial settings^4,5^.

In Korea, PVL-negative ST72-SCC*mec* type IV MRSA is a major CA-MRSA strain^6,7^. The ST72 MRSA strain has also become an important pathogen in hospitals as well as in the community^8–10^. There have been several studies regarding the prevalence and characteristics of ST72 MRSA, but no previous study has analyzed longitudinal changes in ST72 MRSA. We hypothesized that there were changes in the properties of ST72 MRSA and analyzed changes in the clinical characteristics of patients with ST72 MRSA bacteremia as well as changes in microbiological characteristics and the genotype of ST72 MRSA isolates in our institution over 11 years.

## Materials and methods

### Study population and design

This prospective study with a cohort of patients with *S. aureus* bacteremia (SAB) was conducted at the Asan Medical Center, a 2,700-bed tertiary referral center in Seoul, South Korea. From July 2008 to December 2018, all adult patients with SAB were prospectively enrolled in a cohort and observed over a 12-week period. Patients were excluded if they (1) had polymicrobial bacteremia or (2) were discharged before positive blood culture results were available. Of the patients with MRSA bacteremia, only those with ST72 MRSA isolates were included in the study. This study consisted of two sets of analysis; a longitudinal analysis to evaluate changes in the clinical characteristics and outcomes of ST72 MRSA bacteremia, and a comparison of clinical characteristics and outcomes in the first 3 years (2008–2010) and final 3 years (2016–2018). We also evaluated subgroup analysis in patients with community-onset and HA infection. Informed consent was obtained from all individual participants included in the research. All methods were performed in accordance with relevant guidelines and regulations. This study was approved by the Asan Medical Center Institutional Review Board.

### Data collection and study definitions

The following data were recorded: age, gender, demographic characteristics, underlying diseases, severity of underlying disease or condition, bacteremia severity, location of acquisition, initial source of SAB, susceptibility to antibiotics, and clinical outcomes. Acute Physiology and Chronic Health Evaluation II (APACHE II) and Pitt bacteremia scores were used to evaluate the severity of bacteremia, and the Charlson comorbidity index were used to evaluate the severity of comorbid conditions^11–13^.

Location of acquisition was categorized as CA, healthcare-associated, or HA infection^14^. Catheter-related infection and infective endocarditis were defined in accordance with widely accepted criteria^15,16^. Prosthetic devices included orthopedic devices, prosthetic valves, cardiovascular electronic devices, and vascular grafts. Septic shock was defined according to the Third International Consensus Definitions for Sepsis and Septic Shock sepsis^17^. Persistent bacteremia was defined as bacteremia for ≥ 3 d while receiving appropriate antibiotic therapy. Recurrent bacteremia was defined as the occurrence of SAB within 12 weeks following clinical resolution; 30-day mortality and 12-week mortality mean all-cause deaths within 30 days and 12 weeks after bacteremia, respectively. Infection-attributable mortality was death due to ST72 MRSA infection in a previously healthy individual or when ST72 MRSA infection hastened death in the presence of an underlying medical condition.

### Microbiology analysis

The first bloodstream isolates from each patient were used for microbiological and molecular assessments. All isolates were confirmed as MRSA by polymerase chain reaction detection of the *mec*A gene, and multi-locus sequence typing was performed as described elsewhere^18^. The vancomycin minimum inhibitory concentrations (MICs) were determined using a broth microdilution method based on the manufacturer's protocol. Antimicrobial susceptibilities were tested by standard techniques according to the Clinical and Laboratory Standards Institute.

The staphylococcal cassette chromosome *mec* (SCC*mec*) type, *agr* functionality, and *spa* sequence typing were identified using previously described methods^19,20^. The *agr* disfunction was determined by analyzing δ-hemolysin activity as described previously^21^. The assignment of *spa* type was performed using BIONUMERICS (APPLIED MATHS).

### Statistical analysis

All variables for each year's clinical characteristics, *agr* functionality, SCC*mec* type, and *spa* type were summarized, and annual changes were analyzed using a generalized linear model. Continuous variables were analyzed using a linear regression model. We compared patients in the first 3 years (2008–2010) and in the final 3 years (2016–2018). Categorical variables were compared using the Chi-squared test or Fisher’s exact test, as appropriate. Continuous variables were compared using Student's *t* test and the Mann–Whitney U-test. A two-tailed *p* value less than 0.05 was considered significant. All statistical analyses were performed using SPSS software, version 21.0 (IBM Corp., Armonk, NY, USA).

### Ethics approval

This study was approved by the hospital ethics committee.

## Results

### Annual change in clinical characteristics and outcomes of ST72 MRSA

**Figure 1:**
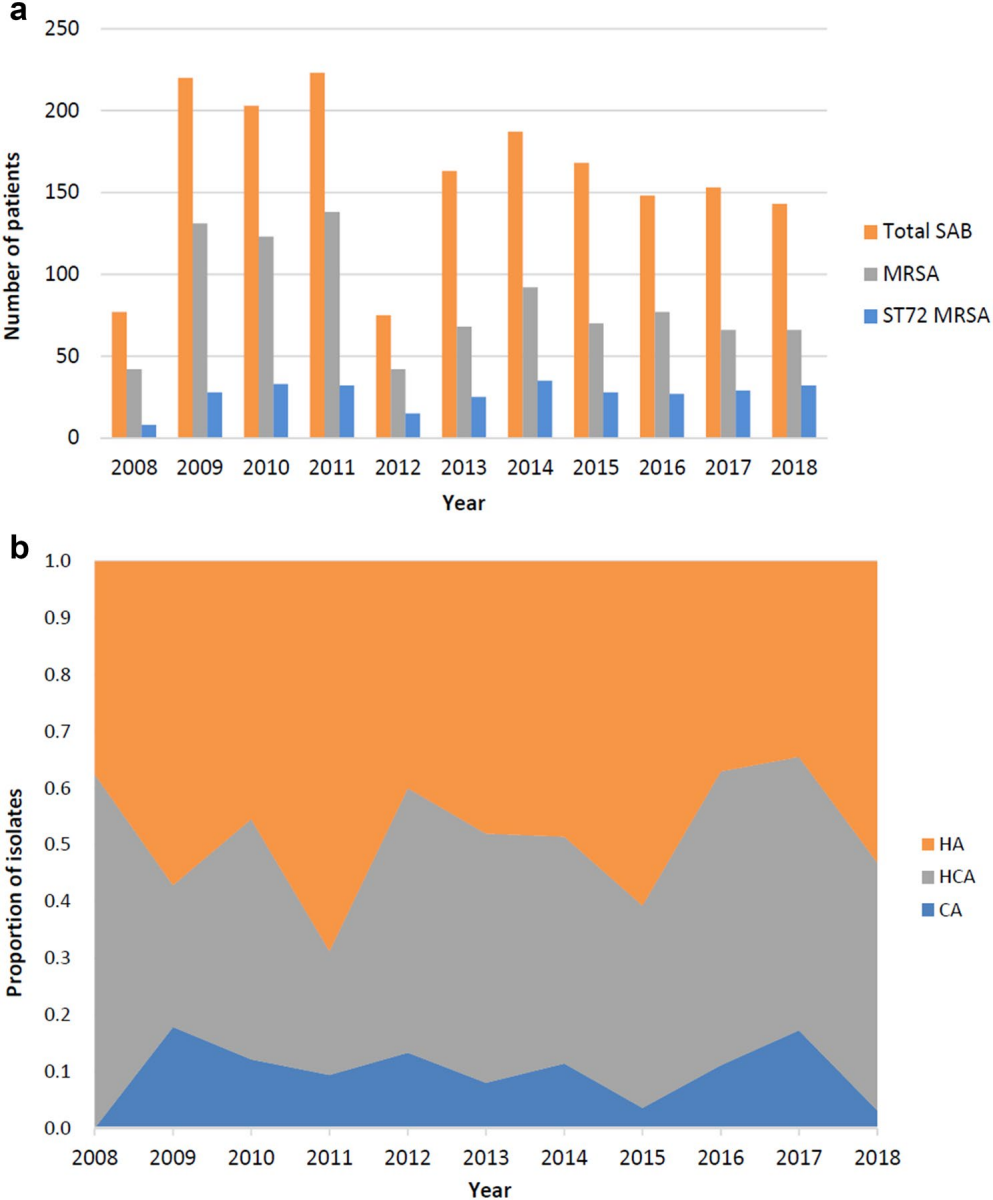
(**a**) Annual distribution of *Staphylococcus aureus* bacteremia (SAB), MRSA, and ST72 MRSA in Asan Medical Center over an 11-year period. (**b**) Locations of acquisition of ST72 MRSA over an 11-year period. *MRSA* methicillin-resistant *Staphylococcus aureus*; *ST* sequence type; *HA* Hospital-acquired; *HCA* healthcare-associated; *CA* community-acquired.

From July 2008 to December 2018, there were 1,760 episodes of SAB, and 915 (62%) were MRSA bacteremia. Among the 915 MRSA isolates, 292 (31.9%) were ST72 MRSA. During the study period, the relative risk (RR) of MRSA bacteremia decreased annually by 3.7% (*p* < 0.01) and among MRSA bacteremia, the RR of ST72 MRSA increased annually by 8.5% (*p* < 0.01) (Fig. 1a). Locations of acquisition of ST72 MRSA over an 11-year period are shown in Fig. 1b. The clinical and microbiological characteristics of ST72 MRSA are shown in Table 1. HA bacteremia was found in 49.7% (145/292) of cases and CA bacteremia in 10.3% (30/292) of cases. Most common comorbidities were diabetes mellitus (37.0% [108/292]), solid tumor (36.6% [107/292]), liver cirrhosis (14.4% [42/292]), and end state renal disease (14.0% [41/292]). The site of acquisition of MRSA bacteremia and underlying diseases did not show significant change during the study period.

**Table 1:** Annual changes in clinical and microbiological characteristics of 292 patients with sequence type 72 MRSA bacteremia.

Characteristic	Number of patients (%)	Annual change	
		RR (95% CI)	*p* value
Age (year), median (IQR)	64 (53–72)	NA	NA
Male	175 (59.9)	NA	NA
**Place of acquisition**			
Community-onset	147 (50.3)	1.02 (0.97–1.08)	0.47
Community-acquired	30 (10.3)	0.96 (0.85–1.08)	0.47
Healthcare-associated	117 (40.1)	1.04 (0.98–1.10)	0.24
Hospital-acquired	145 (49.7)	0.98 (0.93–1.03)	0.47
**Underlying disease**			
Diabetes mellitus	108 (37.0)	1.00 (0.94–1.07)	0.94
Solid tumor	107 (36.6)	1.01 (0.95–1.08)	0.76
Liver cirrhosis	42 (14.4)	1.08 (0.98–1.20)	0.14
End state renal disease	41 (14.0)	1.01 (0.91–1.12)	0.86
Charlson comorbidity index, median (IQR)	2 (2–4.8)	0.05*	0.30
APACHE II, median (IQR)	16 (11–20)	− 0.03*	0.83
Pitt bacteremia score, median (IQR)	1 (0–2)	− 0.01*	0.82
Septic shock	33 (11.3)	0.96 (0.86–1.07)	0.45
Central venous catheter	88 (30.1)	1.00 (0.93–1.07)	0.93
Prosthetic device^a^	58 (19.9)	0.99 (0.91–1.07)	0.75
**Characteristics of infection**			
CVC-related infection	58 (19.9)	0.94 (0.86–1.03)	0.16
Primary bacteremia	42 (14.4)	0.98 (0.89–1.09)	0.72
Bone and joint infection	33 (11.3)	0.96 (0.86–1.08)	0.48
Skin and soft tissue	26 (8.9)	1.08 (0.95–1.23)	0.26
Pneumonia	24 (8.2)	1.17 (1.02–1.35)	0.03
Infective endocarditis	14 (4.8)	1.01 (0.85–1.20)	0.91
Metastatic infection	57 (19.5)	0.89 (0.82–0.97)	0.01
Persistent bacteremia (≥ 3 d)	116 (39.7)	0.94 (0.89–1.00)	0.045
Recurrent bacteremia within 12 weeks	13 (4.5)	0.70 (0.56–0.89)	< 0.01
30-d mortality	34 (11.6)	1.08 (0.97–1.21)	0.17
12-week mortality	65 (22.3)	1.02 (0.94–1.11)	0.60
Infection-attributable mortality	38 (13.0)	1.08 (0.97–1.20)	0.17
In-hospital crude mortality	39 (13.4)	1.05 (0.95–1.17)	0.34
Vancomycin MIC (BMD), mean ± SD (mg/L)	1.08 ± 0.20	− 0.01*^b^	0.01
*agr* dysfunction	31 (10.6)	1.02 (0.90–1.14)	0.79
**SCC*mec* type**			
SCC*mec* IV	291 (99.7)	1.00 (0.96–1.04)	0.96
SCC*mec* IVa	251/291 (86.3)	0.99 (0.95–1.03)	0.64
***spa* type**			
t324	139 (47.6)	0.96 (0.91–1.02)	0.19
t664	42 (14.4)	0.96 (0.87–1.06)	0.39
t148	33 (11.3)	0.98 (0.88–1.10)	0.72
Others	49 (16.8)	NA	NA
**Antibiotic resistance**			
Amoxicillin/Clavulanate	271 (92.8)	1.00 (0.97–1.04)	0.85
Clindamycin	58 (19.9)	0.98 (0.90–1.07)	0.68
Ciprofloxacin	23 (7.9)	1.15 (1.00–1.33)	0.05
Erythromycin	75 (25.7)	0.89 (0.82–0.96)	< 0.01
Fusidic acid	3 (1.0)	1.11 (0.75–1.63)	0.60
Gentamicin	39 (13.4)	0.95 (0.86–1.06)	0.35
Rifampin	5 (1.7)	1.12 (0.83–1.51)	0.47
Quinupristin/Dalfopristin	0	NA	NA
TMP/SMX	2 (0.7)	0.85 (0.53–1.39)	0.52
Tetracycline	8 (2.7)	1.07 (0.85–1.35)	0.58

*IQR* interquartile range; *APACHE II* acute physiology and chronic health evaluation II; *CVC* central venous catheter; *MIC* minimum inhibitory concentration; *BMD* broth microdilution method; *SCC* staphylococcal cassette chromosome; *TMP/SMX* trimethoprim/sulfamethoxazole.

*A linear regression model was used for analysis.

^a^Prosthetic device included orthopedic devices (14 patients), cardiovascular implantable electronic devices (4 patients), prosthetic valves (11 patients), and vascular grafts (30 patients) in patients with persistent bacteremia.

^b^Result of linear regression: R^2^ = 0.025, vancomycin MIC = 22.07 + (− 0.01) × year.

**Figure 2:**
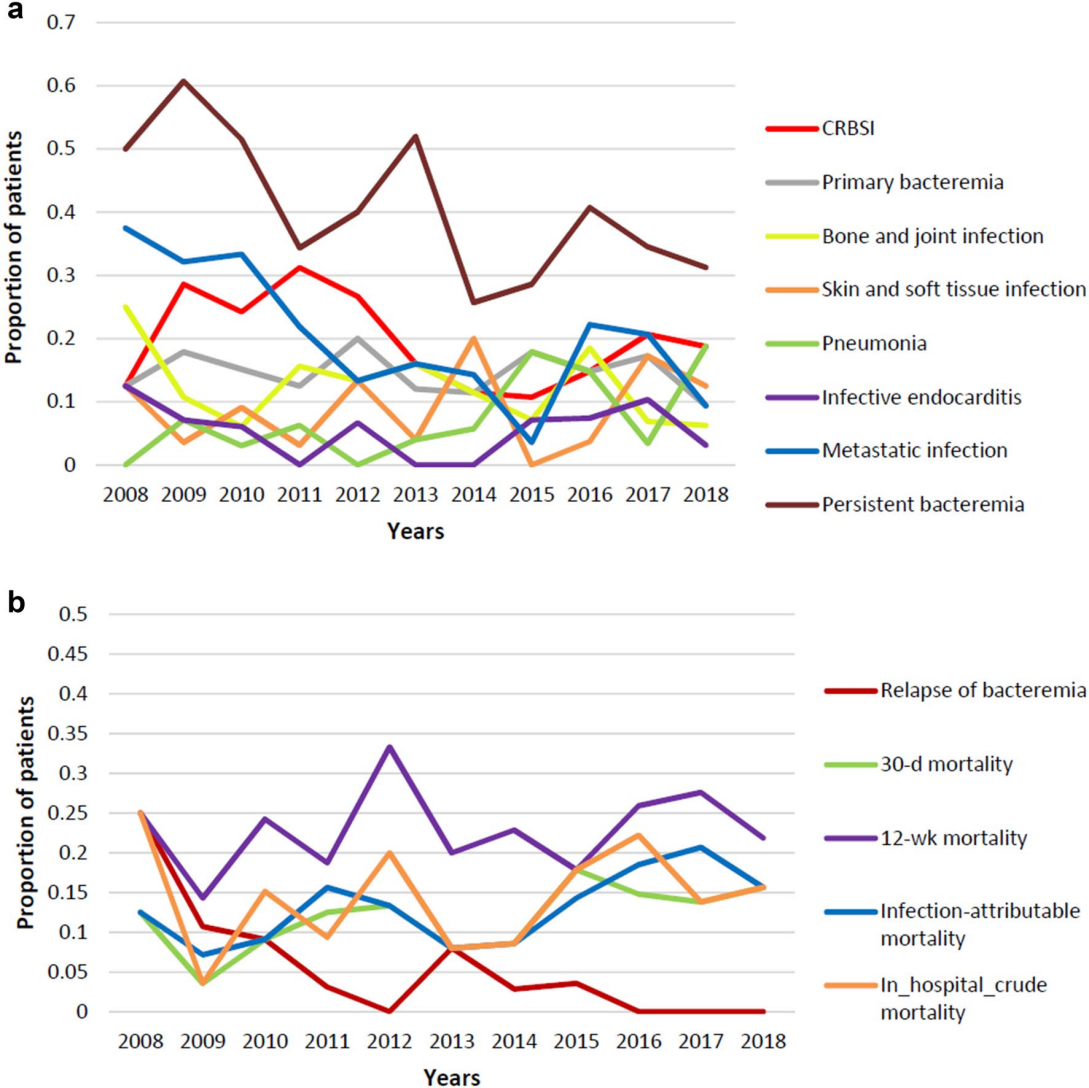
(**a**) Clinical characteristics of ST72 MRSA bacteremia over the study period. (**b**) Clinical outcomes of ST72 MRSA bacteremia over the study period. CRBSI, catheter-related blood stream infection.

There were no significant changes in Charlson comorbidity index, APACHE II score, or Pitt bacteremia score*.* Characteristics of infection were identified as central venous catheter (CVC)-related infection (19.9% [58/292]), primary bacteremia (14.4% [42/292]), bone and joint infection (11.3% [33/292]), skin and soft tissue infection (8.9% [26/292]), pneumonia (8.2% [24/292]), and infective endocarditis (4.8% [14/292]). The RR of pneumonia increased annually by 15.9% (*p* = 0.03) (Fig. 2a).

Metastatic infection was found in 19.5% (57/292) of cases and persistent bacteremia in 39.7% (116/292) of cases. Metastatic infection (RR 0.89; *p* = 0.01) and persistent bacteremia (RR 0.94; *p* = 0.045) showed significant annual decrease (Fig. 2a). During the study period, 34 (11.6%) patients died within 30 d after the index day and 258 (88.4%) patients survived. There were no significant changes in 30-d and 12-week mortality. Recurrent ST72 MRSA bacteremia occurred in 13 (4.5%) cases and decreased over the study period (RR 0.7; *p* < 0.01) (Fig. 2b).

### Annual changes in microbiological characteristics and antibiotic susceptibility of ST72 MRSA

**Figure 3:**
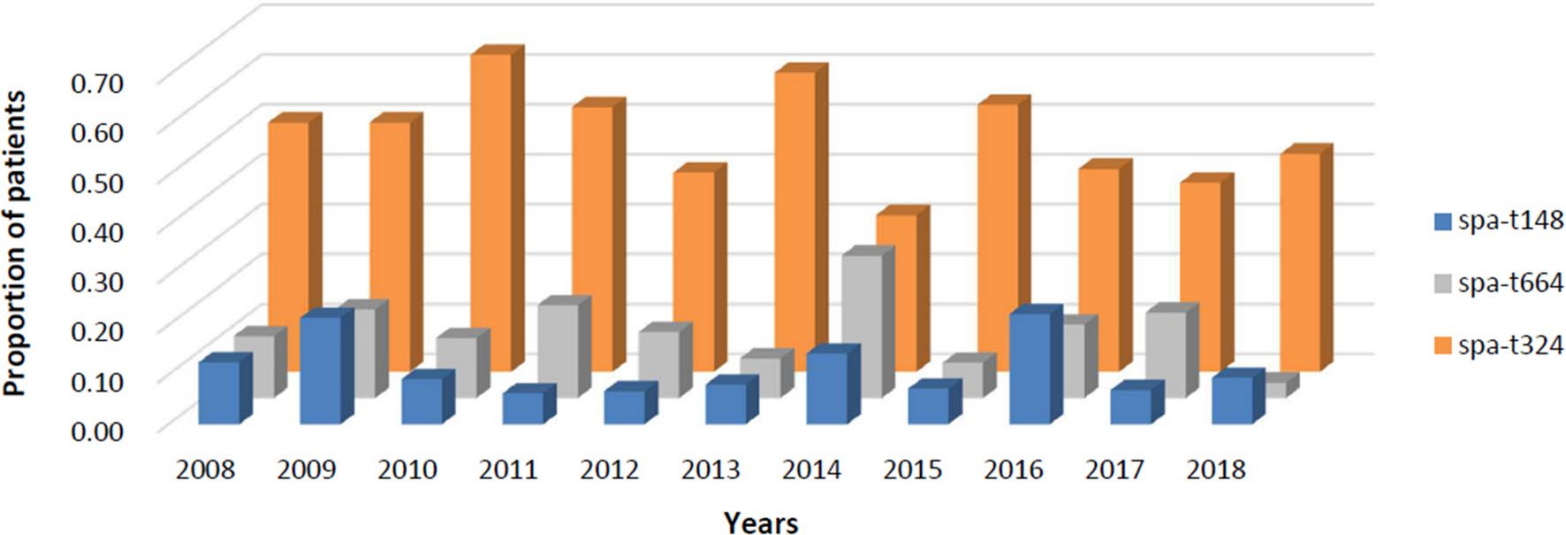
The *spa* type over the study period.

The mean vancomycin MIC was 1.08 mg/L, and this decreased annually by 0.01 (R^2^ = 0.025; *p* = 0.01). *agr* dysfunction was 10.6% (31/292). SCC*mec*IV was 99.7% (291/292), and most SCC*mec*IV was SCC*mec*IVa (86.3%, [251/291]). Common *spa* types were t324 (47.6% [139/292]), t664 (14.4% [42/292]), and t148 (11.3% [33/292]) (Fig. 3). The proportions of *agr* dysfunction, SCC*mec* type, and *spa* type did not differ over the study period. Antibiotic susceptibility tests were conducted on 10 antibiotics. There was no change in the resistance rate to most antibiotics, but that to erythromycin decreased significantly during the study period (RR 0.89; *p* < 0.01).

### Comparison of ST72 MRSA between the first 3 years and final 3 years of study

In the first 3 years (2008–2010), 296 patients had MRSA bacteremia and 69 (23.3%) of these cases were ST72 MRSA. In the final 3 years (2016–2018), 209 patients had MRSA bacteremia and 88 (42.1%) of these cases were ST72 MRSA. The proportions of ST72 MRSA bacteremia was significantly higher in patients in the final 3 years of study than in those in the first 3 years of study (*p* < 0.01). Comparisons of the two groups are shown in Table 2. There were no significant differences in the location of acquisition, underlying disease, and characteristics of infection between the two groups. The patients in the first 3 years were more likely to have metastatic infection (33.3% [23/69] vs. 17.0% [15/88], *p* = 0.02) and persistent bacteremia (55.1% [38/69] vs. 35.2% [31/88], *p* = 0.01) than patients in the final 3 years. Recurrent bacteremia occurred more frequently in patients in the first 3 years (11.6% [8/69] vs. 0% [0/88], *p* < 0.01). There was no difference in 30-d and 12-week mortality between the two groups.

**Table 2:** Univariate analysis of clinical and microbiological characteristics of 157 patients with sequence type 72 MRSA bacteremia.

Characteristic	2008–2010 (n = 69) No. (%)	2016–2018 (n = 88) No. (%)	*p* value
Age (year), median (IQR)	64 (55–72.5)	64.5 (52.3–74)	0.89
Male	36 (52.2)	56 (63.6)	0.15
**Place of acquisition**			
Community-onset	35 (50.7)	51 (58.0)	0.42
Community-acquired	9 (13.0)	9 (10.2)	0.58
Healthcare-associated	26 (37.7)	42 (47.7)	0.21
Hospital-acquired	34 (49.3)	37 (42.0)	0.37
**Underlying disease**			
Diabetes mellitus	30 (43.5)	40 (45.5)	0.81
Solid tumor	25 (36.2)	31 (35.2)	0.90
Liver cirrhosis	8 (11.6)	15 (17.0)	0.34
End state renal disease	10 (14.5)	12 (13.6)	0.88
Charlson comorbidity index, median (IQR)	3 (2–5)	2.5 (1–5)	0.59
APACHE II, median (IQR)	16 (10.5–20)	16 (11–19.8)	0.88
Pitt bacteremia score, median (IQR)	1 (0–2)	0 (0–2)	0.69
Septic shock	9 (13.0)	9 (10.2)	0.58
Central venous catheter	21 (30.4)	28 (31.8)	0.85
Prosthetic device	10 (14.5)	14 (15.9)	0.81
**Characteristics of infection**			
CVC-related infection	17 (24.6)	16 (18.2)	0.32
Primary bacteremia	11 (15.9)	12 (13.6)	0.69
Bone and joint infection	7 (10.1)	9 (10.2)	0.99
Skin and soft tissue	5 (7.2)	10 (11.4)	0.38
Pneumonia	3 (4.3)	11 (12.5)	0.08
Infective endocarditis	5 (7.2)	6 (6.8)	> 0.99
Metastatic infection	23 (33.3)	15 (17.0)	0.02
Persistent bacteremia (≥ 3 d)	38 (55.1)	31 (35.2)	0.01
Recurrent bacteremia within 12 weeks	8 (11.6)	0	< 0.01
30-d mortality	5 (7.2)	13 (14.8)	0.14
12-week mortality	14 (20.3)	22 (25.0)	0.49
Infection-attributable mortality	6 (8.7)	16 (18.2)	0.09
In-hospital crude mortality	8 (11.6)	15 (17.0)	0.34
Vancomycin MIC (BMD), mean ± SD (mg/L)	1.11 ± 0.21	1.03 ± 0.17	< 0.01
*agr* dysfunction	6 (8.7)	8 (9.1)	0.93
***spa* type**			
t324	39 (56.5)	36 (40.9)	0.05
t664	10 (14.5)	10 (11.4)	0.56
t148	10 (14.5)	11 (12.5)	0.72
Others	7 (10.1)	16 (18.2)	NA
**Antibiotic resistance**			
Amoxicillin/Clavulanate	67 (97.1)	81 (92.0)	0.30
Clindamycin	12 (17.4)	14 (15.9)	0.80
Ciprofloxacin	4 (5.8)	10 (11.4)	0.22
Erythromycin	29 (42.0)	14 (15.9)	< 0.01
Fusidic acid	0	1 (1.1)	> 0.99
Gentamicin	11 (15.9)	7 (8.0)	0.12
Rifampin	0	2 (2.3)	0.50
Quinupristin/Dalfopristin	0	0	NA
TMP/SMX	1 (1.4)	0	0.44
Tetracycline	1 (1.4)	2 (2.3)	> 0.99

Vancomycin MIC of patients in the first 3 years was higher than that in patients in the final 3 years (1.11 mg/L vs. 1.03 mg/L, *p* < 0.01). No significant difference was noted in *agr* dysfunction and *spa* type. When comparing the antibiotic resistance of the two groups, the rates of resistance to erythromycin were significantly higher in patients in the first 3 years (42.0% [29/69] vs. 15.9% [14/88], *p* < 0.01).

### Annual changes in clinical characteristics and outcomes of patients with community-onset vs. hospital-acquired infection

We analyzed patients with community-onset and HA infection (Tables 3 and 4). Metastatic infection (RR 0.89; *p* = 0.04) and recurrent bacteremia (RR 0.64; *p* = 0.01) decreased in patients with community-onset infection, but no change was noted in those with HA infection. The resistance rate to erythromycin decreased significantly during the study period (RR 0.87; *p* = 0.01) in patients with community-onset infection but there was no significant change in those with HA infection. Vancomycin MIC decreased annually by 0.01 (R^2^ = 0.032; *p* = 0.03) and resistance rate to ciprofloxacin increased (RR 1.24; *p* = 0.03) in patients with HA infection during the study period.

**Table 3:** Annual changes in the clinical and microbiological characteristics of 147 patients with community-onset infection.

Characteristic	Number of patients (%)	Annual change	
		RR (95% CI)	*p* value
Age (year), median (IQR)	65 (54–73)	NA	NA
Male	92 (62.6)	NA	NA
**Underlying disease**			
Diabetes mellitus	61 (41.5)	0.99 (0.91–1.08)	0.81
Solid tumor	48 (32.7)	0.98 (0.89–1.08)	0.67
Liver cirrhosis	18 (12.2)	1.06 (0.91–1.24)	0.47
End state renal disease	27 (18.4)	0.96 (0.85–1.09)	0.54
Charlson comorbidity index, median (IQR)	2 (1–4)	0.04*	0.56
APACHE II, median (IQR)	15 (11–19)	0.01*	0.97
Pitt bacteremia score, median (IQR)	0 (0–2)	0.01*	0.86
Septic shock	13 (8.8)	0.92 (0.77–1.10)	0.34
Central venous catheter	22 (15.0)	1.03 (0.89–1.18)	0.72
Prosthetic device^a^	37 (25.2)	0.96 (0.86–1.07)	0.43
**Characteristics of infection**			
CVC-related infection	15 (10.2)	0.93 (0.79–1.10)	0.41
Primary bacteremia	19 (12.9)	1.08 (0.93–1.26)	0.34
Bone and joint infection	22 (15.0)	0.96 (0.84–1.10)	0.54
Skin and soft tissue	22 (15.0)	1.06 (0.92–1.22)	0.44
Pneumonia	14 (9.5)	1.08 (0.93–1.26)	0.34
Infective endocarditis	10 (6.8)	0.94 (0.77–1.15)	0.55
Metastatic infection	36 (24.5)	0.89 (0.80–0.99)	0.04
Persistent bacteremia (≥ 3 d)	69 (46.9)	0.94 (0.87–1.02)	0.14
Recurrent bacteremia within 12 weeks	9 (6.1)	0.64 (0.47–0.87)	0.01
30-d mortality	15 (10.2)	1.10 (0.93–1.32)	0.27
12-week mortality	29 (19.7)	1.00 (0.89–1.12)	0.95
Infection-attributable mortality	21 (14.3)	1.05 (0.91–1.21)	0.49
In-hospital crude mortality	15 (10.2)	1.08 (0.91–1.28)	0.39
Vancomycin MIC (BMD), mean ± SD (mg/L)	1.10 ± 0.02	− 0.01*^b^	0.06
*agr* dysfunction	18 (12.2)	0.99 (0.85–1.15)	0.85
**SCC*mec* type**			
SCC*mec* IV	147 (100.0)	1.00 (0.95–1.05)	> 0.99
SCC*mec* IVa	129/147 (87.8)	0.99 (0.94–1.05)	0.81
***spa* type**			
t324	67 (45.6)	0.96 (0.89–1.04)	0.35
t664	20 (13.6)	0.99 (0.86–1.14)	0.90
t148	19 (12.9)	0.99 (0.86–1.15)	0.93
Others	25 (17.0)	NA	NA
**Antibiotic resistance**			
Amoxicillin/Clavulanate	139 (94.6)	1.00 (0.95–1.06)	0.97
Clindamycin	27 (18.4)	0.99 (0.87–1.12)	0.86
Ciprofloxacin	10 (6.8)	1.06 (0.86–1.30)	0.60
Erythromycin	38 (25.9)	0.87 (0.78–0.96)	0.01
Fusidic acid	1 (0.7)	NA	NA
Gentamicin	25 (17.0)	0.92 (0.81–1.10)	0.23
Rifampin	3 (2.0)	1.24 (0.80–1.93)	0.34
Quinupristin/Dalfopristin	0	NA	NA
TMP/SMX	1 (0.7)	NA	NA
Tetracycline	4 (2.7)	1.02 (0.74–1.41)	0.91

*A linear regression model was used for analysis.

^a^Prosthetic device included orthopedic devices (8 patients), cardiovascular implantable electronic devices (1 patients), prosthetic valves (5 patients), and vascular grafts (24 patients) in patients with persistent bacteremia.

^b^Result of linear regression: R^2^ = 0.025, vancomycin MIC = 22.07 + (− 0.01) × year.

**Table 4:** Annual changes in clinical and microbiological characteristics of 145 patients with hospital-acquired infection.

Characteristic	Number of patients (%)	Annual change	
		RR (95% CI)	*p* value
Age (year), median (IQR)	62 (49.5–70.5)	NA	NA
Male	83 (57.2)	NA	NA
**Underlying disease**			
Diabetes mellitus	47 (32.4)	1.01 (0.92–1.11)	0.79
Solid tumor	59 (40.7)	1.04 (0.96–1.13)	0.37
Liver cirrhosis	24 (16.6)	1.10 (0.96–1.26)	0.16
End state renal disease	14 (9.7)	1.09 (0.91–1.30)	0.34
Charlson comorbidity index, median (IQR)	2 (2–5)	0.06*	0.36
APACHE II, median (IQR)	16 (11–21)	− 0.04*	0.85
Pitt bacteremia score, median (IQR)	1 (0–2)	− 0.02*	0.74
Septic shock	20 (13.8)	0.99 (0.86–1.15)	0.92
Central venous catheter	66 (45.5)	1.00 (0.92–1.08)	0.99
Prosthetic device^a^	21 (14.5)	1.02 (0.89–1.18)	0.75
**Characteristics of infection**			
CVC-related infection	43 (29.7)	0.96 (0.86–1.06)	0.38
Primary bacteremia	23 (15.9)	0.91 (0.79–0.15)	0.19
Bone and joint infection	11 (7.6)	0.95 (0.78–1.16)	0.59
Skin and soft tissue	4 (2.8)	1.09 (0.79–1.52)	0.61
Pneumonia	10 (6.9)	1.21 (0.97–1.52)	0.09
Infective endocarditis	4 (2.8)	1.19 (0.84–1.68)	0.32
Metastatic infection	21 (14.5)	0.88 (0.76–1.03)	0.11
Persistent bacteremia (≥ 3 d)	47 (32.4)	0.93 (0.84–1.02)	0.12
Recurrent bacteremia within 12 weeks	4 (2.8)	0.84 (0.59–1.20)	0.34
30-d mortality	19 (13.1)	1.07 (0.92–1.24)	0.37
12-week mortality	36 (24.8)	1.05 (0.94–1.17)	0.41
Infection-attributable mortality	17 (11.7)	1.11 (0.94–1.30)	0.22
In-hospital crude mortality	24 (16.6)	1.05 (0.92–1.19)	0.51
Vancomycin MIC (BMD), mean ± SD (mg/L)	1.06 ± 0.01	− 0.01*^b^	0.03
*agr* dysfunction	13 (9.0)	1.05 (0.88–1.26)	0.57
**SCC*mec* type**			
SCC*mec* IV	144 (99.3)	1.00 (0.95–1.06)	0.95
SCC*mec* IVa	122/144 (84.7)	0.99 (0.93–1.05)	0.65
***spa* type**			
t324	72 (49.7)	0.97 (0.89–1.04)	0.37
t664	22 (15.2)	0.93 (0.80–1.07)	0.30
t148	14 (9.7)	0.95 (0.80–1.14)	0.60
Others	24 (16.6)	NA	NA
**Antibiotic resistance**			
Amoxicillin/Clavulanate	132 (91.0)	1.01 (0.95–1.07)	0.78
Clindamycin	31 (21.4)	0.98 (0.87–1.01)	0.73
Ciprofloxacin	13 (9.0)	1.24 (1.02–1.52)	0.03
Erythromycin	37 (25.5)	0.91 (0.82–1.02)	0.10
Fusidic acid	2 (1.4)	1.16 (0.72–1.86)	0.56
Gentamicin	14 (9.7)	0.99 (0.83–1.18)	0.92
Rifampin	2 (1.4)	0.98 (0.61–1.55)	0.92
Quinupristin/Dalfopristin	0	NA	NA
TMP/SMX	1 (0.7)	NA	NA
Tetracycline	4 (2.8)	1.12 (0.80–1.57)	0.50

*A linear regression model was used for analysis.

^a^Prosthetic device included orthopedic devices (six patients), cardiovascular implantable electronic devices (three patients), prosthetic valves (six patients), and vascular grafts (six patients) in patients with persistent bacteremia.

^b^Result of linear regression: R^2^ = 0.032, vancomycin MIC = 21.57 + (− 0.01) × year.

## Discussion

In this study, we evaluated the clinical, microbiological, and genotypic changes in ST72 MRSA bacteremia over 11 years. The RR of MRSA bacteremia decreased and among these, the RR of ST72 MRSA increased during the study period. *S. aureus* pneumonia increased. Metastatic infection, persistent bacteremia, and recurrent bacteremia decreased during the study period. The rate of erythromycin resistance decreased significantly over the 11 years.

PVL-negative ST72 MRSA is a major CA-MRSA strain in South Korea and is an important cause of nosocomial infection. The incidence of infections caused by ST72 MRSA is increasing in both community and healthcare settings. Kim et al.^6^ reported that ST72 MRSA isolates constituted 34.7% of CA-MRSA infection and 4.2% of HA-MRSA infection. In blood stream infection, 22.5–25.0% of MRSA bacteremia were ST72 MRSA and 21.0% of HA-MRSA bacteremia were ST72 MRSA^22,23^. In this study, MRSA bacteremia levels decreased over 11 years, but ST72 MRSA increased significantly. ST72 MRSA levels of patients were significantly higher in the final 3 years than in the first 3 years. A large proportion of ST72 MRSA bacteremia were HA infections.

Previous studies have reported conflicting results regarding the effect of ST72 MRSA on the clinical outcome of MRSA infection^24,25^. Park et al.^23^ reported that ST72-SCC*mec*IV was associated with reduced mortality compared with ST5-SCC*mec*II. In this study, 30-d and 12-week mortality did not differ over the study period. CVC-related infection, bone and joint infection, and infective endocarditis have been suggested as risk factors for bacteremia complication^26–28^. In the present study, there were no changes in CVC-related infection, bone and joint infection, and infective endocarditis, but the incidence of bacteremia complications, such as metastatic infection, persistent bacteremia, and recurrent bacteremia, decreased. In subgroup analysis, metastatic infection and recurrent bacteremia only decreased in community-onset infection. These results suggest changes in community-onset MRSA, and further research is warranted.

A high vancomycin MIC is associated with worse clinical outcomes and treatment failure^29–31^. Rybak et al.^32^ reported an increase in the vancomycin MIC of *S. aureus* from 1986 to 2007. The cell wall-active antibiotics inhibit bacterial growth by inhibiting peptidoglycan biosynthesis^33^. Molecular events of bacteria occur after treatment with cell wall-active antibiotics^34,35^. *S. aureus* activates a protective cell wall stress stimulons in response to the inhibition of cell envelope damage or cell wall synthesis caused by several antibiotics^34,36,37^. These factors probably increase the vancomycin MIC. In this study, the vancomycin MIC decreased over the 11-year study period, and the change was significant in HA infection. This may be due to the difference in the study period or study lesions. Additionally, there may be changes in microbiological and genomic factors of *S. aureus*. Further studies are necessary to assess changes in the microbiological and genomic factors of *S. aureus* that are associated with vancomycin MIC.

In Korea, resistance rates to clindamycin, erythromycin, ciprofloxacin, and gentamicin were lower in ST72-SCC*mec*IV MRSA than in ST5-SCC*mec*II MRSA strain^6,9,23^. The resistance rate of ST72 MRSA to erythromycin was from 35.4% to 60.0%^9,23^. In this study, the erythromycin resistance rate of ST72 MRSA decreased over the 11-year study period significantly. The resistance rate of ST72 MRSA in the final 3 years was significantly lower than ST72 MRSA in the first 3 years (15.9% vs. 42.0%, *p* < 0.01). In subgroup analysis, the erythromycin resistance rate of ST72 MRSA decreased only in community-onset infection. Several mechanisms cause erythromycin resistance to *S. aureus*; the presence of multicomponent macrolide efflux pumps (*msr*A, *msr*B) and enzymatic modification (EreA, EreB) of the antibiotics by enzymes^38–41^. In addition, macrolide resistance genes are present in erythromycin-resistant *S. aureus*^42^. Reduced use of macrolides or altered resistance genes of ST72 MRSA may contribute to decreased erythromycin resistance. Further studies are needed regarding changes in the mechanisms of erythromycin resistance.

This study has several limitations. First, this was a single-center study and our findings cannot be generalized to South Korea. Second, we evaluated ST72 MRSA types only, but analysis with other MRSA types, such as ST5 and ST239, may be helpful for understanding changes in *S. aureus* in Korea. However, this was the first large-scale study to analyze longitudinal changes in ST72 MRSA in Korea. This study will benefit future studies in their analysis of changes in *S. aureus* in community and nosocomial environments.

In conclusion, ST72 MRSA is an important pathogen in both community and nosocomial settings and its incidence increased over the 11-year study period. Bacteremia complication, vancomycin MIC, and resistance rate to erythromycin decreased, and these results suggest changes in the characteristics of ST72 MRSA. Further studies, including genome-wide studies, are needed to understand the reasons for changes of ST72 MRSA.

## Data Availability

The data of this study are available by contacting the corresponding author upon reasonable request.
